# Photothermal and gene therapy combined with immunotherapy to gastric cancer by the gold nanoshell-based system

**DOI:** 10.1186/s12951-019-0515-x

**Published:** 2019-07-05

**Authors:** Jiayu Zhang, Tiancheng Zhao, Fanglei Han, Yu Hu, Yezhou Li

**Affiliations:** 1 Department of Vascular Surgery, China-Japan Union Hospital of Jilin University, No. 126 XianTai Street, Changchun, 130033 Jilin China; 2 Department of Gastrointestinal Colorectal and Anal Surgery, China-Japan Union Hospital of Jilin University, No. 126 XianTai Street, Changchun, 130033 Jilin China; 3 Department of Endoscopic Center, China-Japan Union Hospital of Jilin University, No. 126 XianTai Street, Changchun, 130033 Jilin China; 4 Pathology Department, China-Japan Union Hospital of Jilin University, No. 126 XianTai Street, Changchun, 130033 Jilin China; 5 Department of Anesthesiology, China-Japan Union Hospital of Jilin University, No. 126 XianTai Street, Changchun, 130033 Jilin China

## Abstract

**Background:**

The gastric cancer is the second most malignant tumor in the world. HER-2 is one of the key targets for the gastric cancer therapy. Anti-HER-2 antibodies like trastuzumab, exhibits the satisfactory therapeutic effect in clinical. However, the drug resistance problem limits its application.

**Method:**

In this study, we develop a gold nanoshell (Gold Nanoshell) drug carrier for delivery and selective photo-thermal release of genes which target HER-2 and immunologic adjuvant CPG sequence in gastric tumor cells. The drug delivery system generated a multidimensional treatment strategy which includes gene-, immune- and photothermal-therapy.

**Results:**

The whole gold nanoshell drug delivery system exhibits the well gene transduction ability and combined treatment effect. Both in vitro and in vivo results demonstrate the multiple therapeutic effects of the drug delivery system is better than the monotherapy.

**Conclusions:**

This study indicates the multiple combined therapy based on the gold nanoshell system would be a promising translational treatment for gastric cancer.

## Introduction

The gastric cancer is the second most malignant tumor in the world, which ranks after lung cancer. As the unobvious clinical symptoms in the early time, most patients are always diagnosed in late stage. The overall 5-year survival rate is only about 30%, which has become a major threaten to public health [1–5]. Chemotherapy is still as a major means to treat gastric cancer in clinics. But due to the lake of selectivity, chemotherapy also brings serious side effects to the patients [6, 7].

Human epidermal growth factor receptor-2 (HER-2) is one of the most important targets of gastric cancer therapy in the current study [8]. About 7–43% of gastric cancer patients have the HER-2 gene mutation or overexpression [9, 10]. Overexpressed HER-2 activates the growth signal transduction pathway, which indicates it could be an oncogene driver in gastric cancer cells. Directly inhibiting HER-2 or its downstream signal transduction pathways can be used for the treatment of HER-2 positive gastric cancer patients. Trastuzumab, a humanized monoclonal anti-HER-2 antibody, aiming at the extracellular domain of HER-2 and blocking HER-2 to form allodimer to inhibit tumor, which has been approved to treat the HER-2 positive gastric cancer patients [11–13]. The application of trastuzumab has benefited patients with advanced HER-2 positive gastric cancer, but the clinical treatment indicates that most patients have trastuzumab resistance in a relatively short period of time (the median progression free survival was 6.7 months). However, the mechanism of resistance is still not clear. The trastuzumab may induce mutations of HER-2 kinase and increase of HER-2 gene copy number during the treatment, resulting in the occurrence of drug resistance [14–17].

Gene inhibition for the treatment target could effectively suppress the protein expression and improve the therapeutic efficacy [18]. Moreover, the occurrence of secondary drug resistance caused by mutations of the target protein could be avoided in gene therapy. In the gene therapy, the gene fragments are uptaken by the cancer cells in a particular way and the target mRNA is suppressed for inhibiting the corresponding protein expression [19, 20]. Therefore, siRNA (small interfering RNA) targeting HER-2 mRNA is a promising strategy for cancer therapy. In gene therapy, specific techniques or carriers for gene transfection are required to introduce foreign genes into cells. Among the most challenging part of gene carriers are the safety and effectiveness, that is to say, the transfection carriers are required to transfer genes into different tissues efficiently with free of pathogenicity. It is suggested that the success of gene therapy, at least partially, depends on the safety of gene carrier [21–25].

Gold nanoshell is a novel type of nano-carrier which features a spherical core structure with about 30 nm in particle size and good optical-to-thermal conversion efficiency. Gold nanoshell could absorb 785 nm near-infrared light and convert it into thermal energy for in vivo tumor hyperthermia. It is a fairly efficient siRNA nano-carrier with many advantages. Due to the large specific surface area, higher drug loading ability is equipped on the gold nanoshells. At the same time, the unique spatial structure can effectively prevent the siRNA sequence from easily degrading by the enzyme in vivo. Compared with other nanomaterials, gold nanoshells have good biocompatibility and low cytotoxicity [26–28]. There is also a prominent thermal effect on gold nanoparticles after near infrared irradiation. The high temperature breaks the thiol bond, which can trigger the subsequent release of gene therapy agents. Concurrently, the heat released by the gold nanoparticles could damage the tumor cells [29–33].

It is known that photothermal therapy could not only eradicate the treated primary tumors but also induce a systemic antitumor immunity. Laser-induced tumor cell death, on the other hand, can release tumor antigens into the surrounding tissue. At the same time, immunoadjuvants for cancer immunotherapy promote antigen uptake and presentation of antigen-presenting cells, to trigger specific antitumor immunity. Thus one promising strategy is to combine photothermal therapy with immunotherapy. Photothermal therapy may act synergistically with immunotherapy to enhance immune responses.

Oligeoxynucletides containing cytosine–guanine (CpG) motifs as the immunoadjuvant, which act as an efficient modulator for cancer immunotherapy. They can specifically activate Toll-like receptor 9 signaling in plasmacytoid dendritic cell to induce immune response. However, clinical use of CpG oligodeoxynucleotides faces several challenges including in vivo instability, biodistribution characteristics and a requirement for intracellular uptake because Toll-like receptor 9 is located in the endosomal compartment. So how to transport the CpG into Toll-lie receptor 9-rich endosomes of plasmacytoid dendritic cell is a problem to be solved [34–37].

In this study we established a drug delivery system based on the gold nanoshell siRNA@CpG@Gold Nanoshell which colligated gene therapy, hyperthermia and immunotherapy to cope with the challenge of effective gastric cancer treatment. HER-2 as the gene therapy target, exhibited the treatment effect on the molecular level. Concomitantly the photothermal treatment ablated the tumor cells in macroscopic and at last the immunotherapy could trace and kill the cancer cells in the whole body. The all treatments exhibited the well synergistic effect and low side effect. As the drug carrier system, siRNA@CpG@Gold Nanoshell showed many remarkable abilities, such as high drug capacity, siRNA sequence protection, and high thermal conversion efficiency. Via the combinatory treatment, our results have determined that siRNA@CpG@Gold Nanoshell drug carrier system exhibited encouraging antitumor efficacy to against gastric cancer.

## Materials and methods

### Materials

Chloroauric acid, cobalt chloride hexahydrate, trisodium citrate dehydrate, sodium borohydride, glutathione and dithiothreitol were purchased from Sigma Sigma-Aldrich Inc (Shanghai, China). Sulfydryl polyethylene glycol was purchased from Shanghai Sangon Biotech, Inc. Mouse forestomach carcinoma cell (MFC) was obtained from Shanghai cell bank, Chinese academy of sciences (Shanghai, China). Nude and Balb/c mice were purchased from Laboratory Animal Co. Ltd. (ShenYang, China). Bovine serum albumin and cell culture media were purchased from Sangon Biotech (Shanghai) Co., Ltd. The siRNAs and nonsense sequences were purchased from Sangon Biological Engineering Technology & Co. Ltd. (Shanghai, China). The company also accomplished the modifcation of siRNAs and nonsense sequences with -SH and FITC.

### Synthesis and characterization of siRNA@CpG@Gold Nanoshell drug delivery system

Stable suspensions of silver nanoparticles were synthesized in minutes using aqueous solution of sodium citrate and silver nitrate, and the addition of sodium borohydride accelerates the chemical reactions. The reaction proceeded in 60 °C. Larger silver particles can be formed from these nanoparticles by reducing additional silver nitrate in the presence of hydroxylamine hydrochloride; the nanoparticles act as nuclei for further growth. Then the silver nanoparticles were converted into hollow gold nanoshells (Gold Nanoshell) by adding HAuCl4 directly to the as-grown nanoparticle solution and the silver were oxidized and reduced (Ag^+^/Ag 0.8 V, versus a standard hydrogen electrode has a lower redox potential than gold AuCl_4_^−^/Au 0.99 V, the replacement reaction is: $3 {\text{Ag}}_{{({\text{s}})}} + {\text{AuCl}}^{ - }_{{ 4({\text{aq}})}} = {\text{Au}}_{{({\text{s}})}} + 3 {\text{Ag}}^{ + }_{{({\text{aq}})}} + 4 {\text{Cl}}^{ - }_{{({\text{aq}})}}$). Subsequently, multiple copies of siRNA were conjugated to the surface of the Gold Nanoshell either directly or supported by a single strand DNA linker through a quasi-covalent (Au–S) bond (1.5 nM gold nanoshell/200 nM siRNA and 200 nM CpG). Finally, siRNA@CpG@Gold Nanoshell drug delivery system formed and was stored in 4 °C. Morphology and size of the prepared nanoshells were characterized using a JEOL JEM-2100 high-resolution transmission electron microscope (HR-TEM). The hydrodynamic size and zeta-potential of the siRNA@DOX@Gold Nanoshell were measured by Zetasizer (Brookhaven Instruments Corporation, USA) at a temperature of 25 °C.

### Real-time polymerase chain reaction

Total RNA was extracted by using Trizol reagent (Invitrogen), and the reverse-transcription reactions were performed using an M-MLV Reverse Transcriptase kit (Invitrogen). The gene-specific stem–loop RT primers for miRNAs, which were released by the Sanger Institute, were designed according to Chen et al. Real-time PCR was performed using a standard SYBR Green PCR kit (Toyobo, Osaka, Japan) and a Rotor-Gene RG-3000A (Corbett Life Science, Sydney, New South Wales, Australia) according to the instructions from the respective manufacturer. GAPDH was used as references for miRNAs and RNAs, respectively. Each sample was analyzed in triplicate. The 2DDCt method was used to quantify the relative levels of gene expression.

### Western blot

Western blot analysis was carried out on 10% SDS–PAGE. Briefly, HER-2 proteins were electrotransferred onto nitrocellulose filter. After blocking for 1 h in PBS with 0.1% Tween 20 (PBST) and 5% skim milk, the membranes were incubated overnight with specified primary antibody in PBST containing 5% BSA. The primary antibodies used included HER-2 (Cell Signaling Technology) and GAPDH (Bioworld Technology). Assay was carried out by the use of second antibody and developing agent. Western blot was scanned and band intensity was quantified by using Quantity One Imaging Software from Bio-Rad.

### MTT assay

MFC cells were seeded in 96-well plates and cultured for 24 h before exposure to different drugs (with or without irradiation) for 48 h. MTT was then added to each well, and the cells were incubated for 4 h at 37 °C before addition of DMSO. The absorbance was measured at 490 nm with a Miroplate Reader (BioRad).

### Cell apoptosis assay

MFC cells were cultured in 6-well plates overnight and treated with the drug carrier system siRNA@CpG@Gold Nanoshell at indicated doses (1.5 mol/ml) (with or without irradiation) for 48 h. Then cells were collected by centrifugation and stained with annexin V and PI following the manufacturer’s procedure of the Apoptosis Detection Kit (Beyotime Biotechnology). Binding of annexin V and PI to cells was measured by flow cytometry or laser confocal scanning microscopy (LCSM).

### Nucleic acid electrophoresis assay of siRNA protection

To investigate the protective effect of siRNA@Gold Nanoshell, such delivery vectors were incubated with 0.25% RNase A for 0.5 h. Then the sample was loaded on a 1% agarose gel containing 0.01% Gel-Red and run at 90 V for 30 min. To release out the loaded siRNA, the samples were treated with heparin at the dose of 1 U/mg siRNA in DEPC-treated water.

### In vivo imaging of mice

Athymic nude mice were purchased from Laboratory Animal Co. Ltd. (ShenYang, China). MFC cells (5 × 10^6^) were subcutaneously injected into the upper left axillary fossa of nude mice. When the tumors reached 0.5 cm in diameter, mice were used for NIR imaging. The NIR dye ICG (Indocyanine green) adhered to the siRNA@CPG@Gold Nanoshell drug delivery system by electrostatic adsorption. Mice were injected with ICG@siRNA@CPG@ Gold Nanoshell and ICG respectively at a dye dose of 10 mg/kg (5 μl/g of mouse body weight) and then imaged at various time post-injection using an NIR imaging system.

### Therapeutic effect in vivo

Balb/c mice bearing MFC-derived tumors were randomly assigned to 1 control group and 5 experimental groups (n = 5) when the tumor diameter reached 0.5 cm. Mice in the control group were injected with PBS and while the experimental groups were injected with bare nanoshells, siRNA@Gold Nanoshell and siRNA@CPG@Gold Nanoshell (1.5 nM/200 μl gold nanoparticle). 12 h post-injection, different therapeutic schedules were performed on each experimental group: (1) bare nanoshells injection with 785 nm light irradiation (1.2 W/cm^2^) for 5 min; (2) siRNA@Gold Nanoshell injection with 785 nm light irradiation (1.2 W/cm^2^) for 5 min; (3) siRNA@CPG@Gold Nanoshell injection with 785 light irradiation (1.2 W/cm^2^) for 5 min. Tumor sizes and body weights were measured every other day. Tumor volumes were measured using calipers and calculated according to the following formula: size (mm^3^) = length (mm) × width (mm)^2^/2. All mice were sacrificed and the tumors were collected 14 days after treatment.

## Results and discussion

### Synthesis and characterization of siRNA@CPG@Gold Nanoshell

**Scheme 1:**
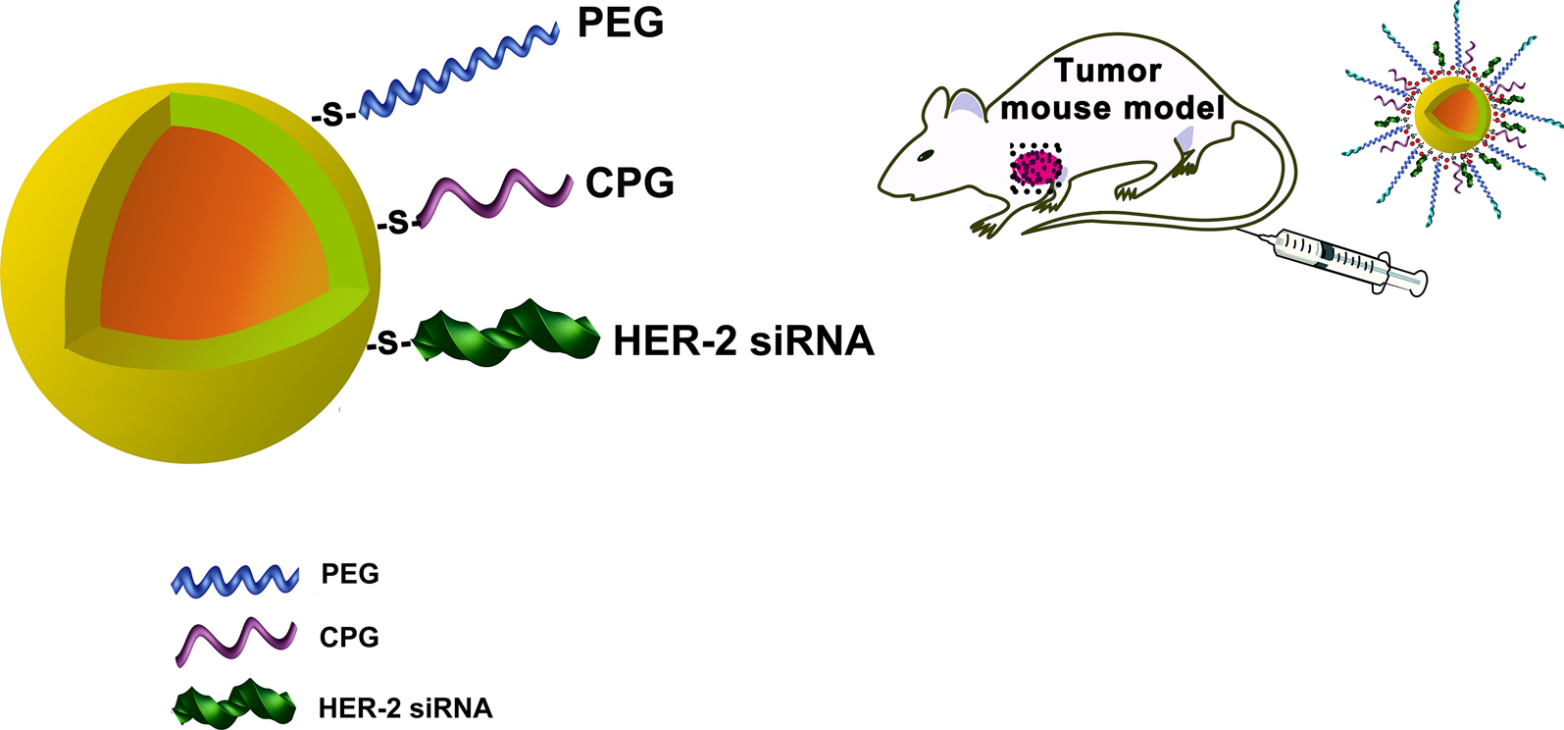
Schematic illustration of the Gold Nanoshell drug carrier system design

**Fig. 1:**
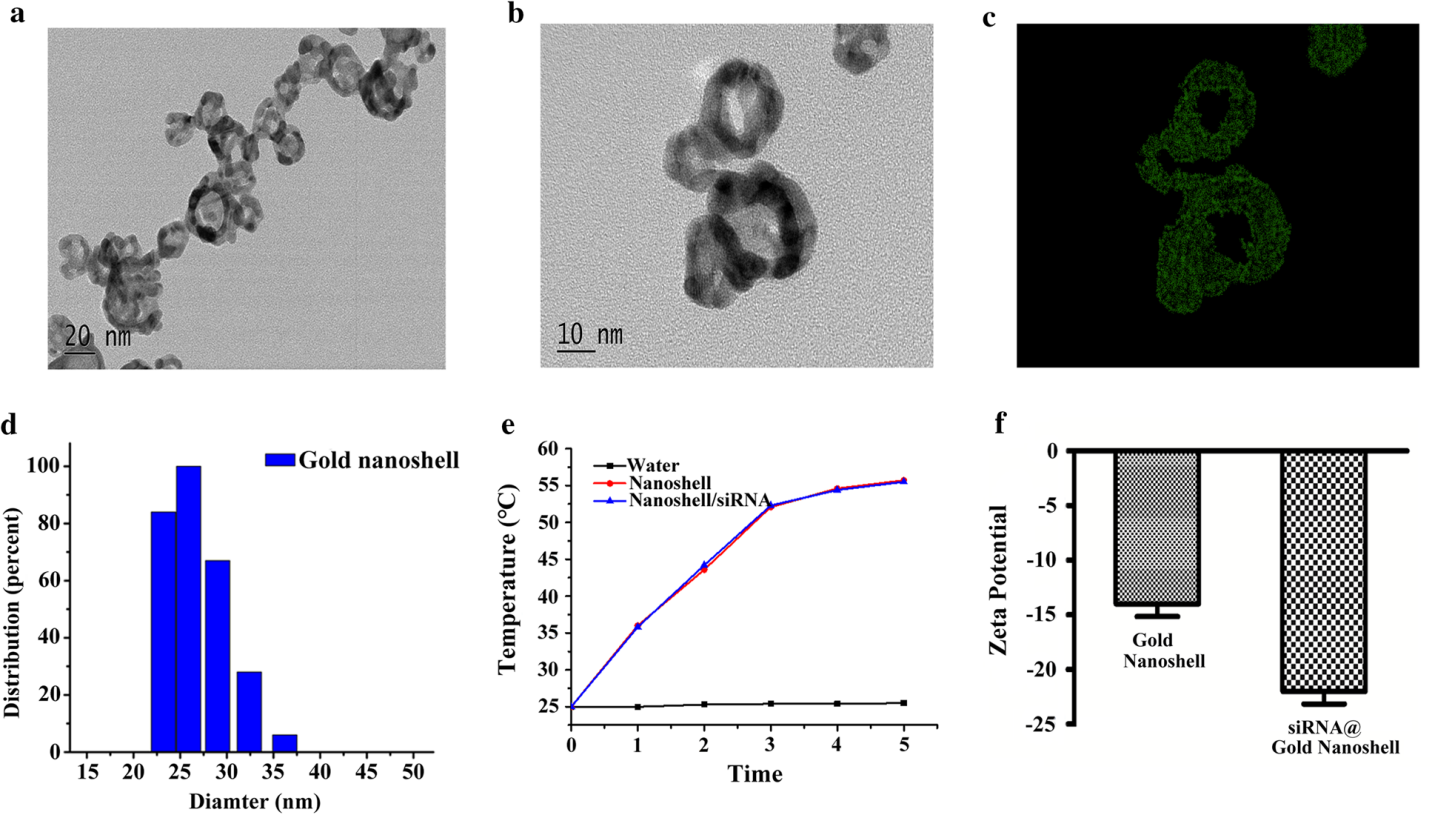
Charaterization of siRNA@CPG@Gold Nanoshell system. **a** Transmission electron microscope (TEM) image of siRNA@CPG@Gold Nanoshell system. **b**, **c** Elemental mapping of Gold nanoshell. **d** The dynamic light scattering (DLS) of siRNA@CPG@Gold Nanoshell system. **e** The photothermal conversion of Gold Nanoshell and siRNA@Gold Nanoshell with laser irradiation. **f** Zeta potential of Gold Nanoshell and siRNA@Gold Nanoshell. Data are given as mean ± SD (n = 5). *, P < 0.05

The structure of siRNA@CPG@Gold Nanoshell is illustrated in Scheme 1. The transmission electron microscopy (TEM) image of the prepared siRNA@CPG@Gold Nanoshell is loaded in Fig. 1a which shows a core–shell structure and the graph reveals that the particle size distribution of drug carrier is uniform. And the elemental mapping is showed in Fig. 1b, c which suggests the nanoshell is constituted by gold. Then, hydrated particle diameter is measured by a laser particle size analyzer, the mean diameter of the drug system is around 30 nm (Fig. 1d). The photothermal conversion study has determined that laser irradiation can quickly and significantly increase temperature from 25 to ~ 56 °C in 5 min (Fig. 1e). However, there is a change in the zeta potential during the drug system synthesis process (Fig. 1f). The Zeta potential of Gold Nanoshell is − 14, and it changes to − 22 after uploading the negatively charged siRNA. The change in potential indicates the success of each step reaction and the production. Till now we get the novel drug delivery system siRNA@CPG@Gold Nanoshell.

### The chemical and drug release property of siRNA@CPG@Gold Nanoshell

**Fig. 2:**
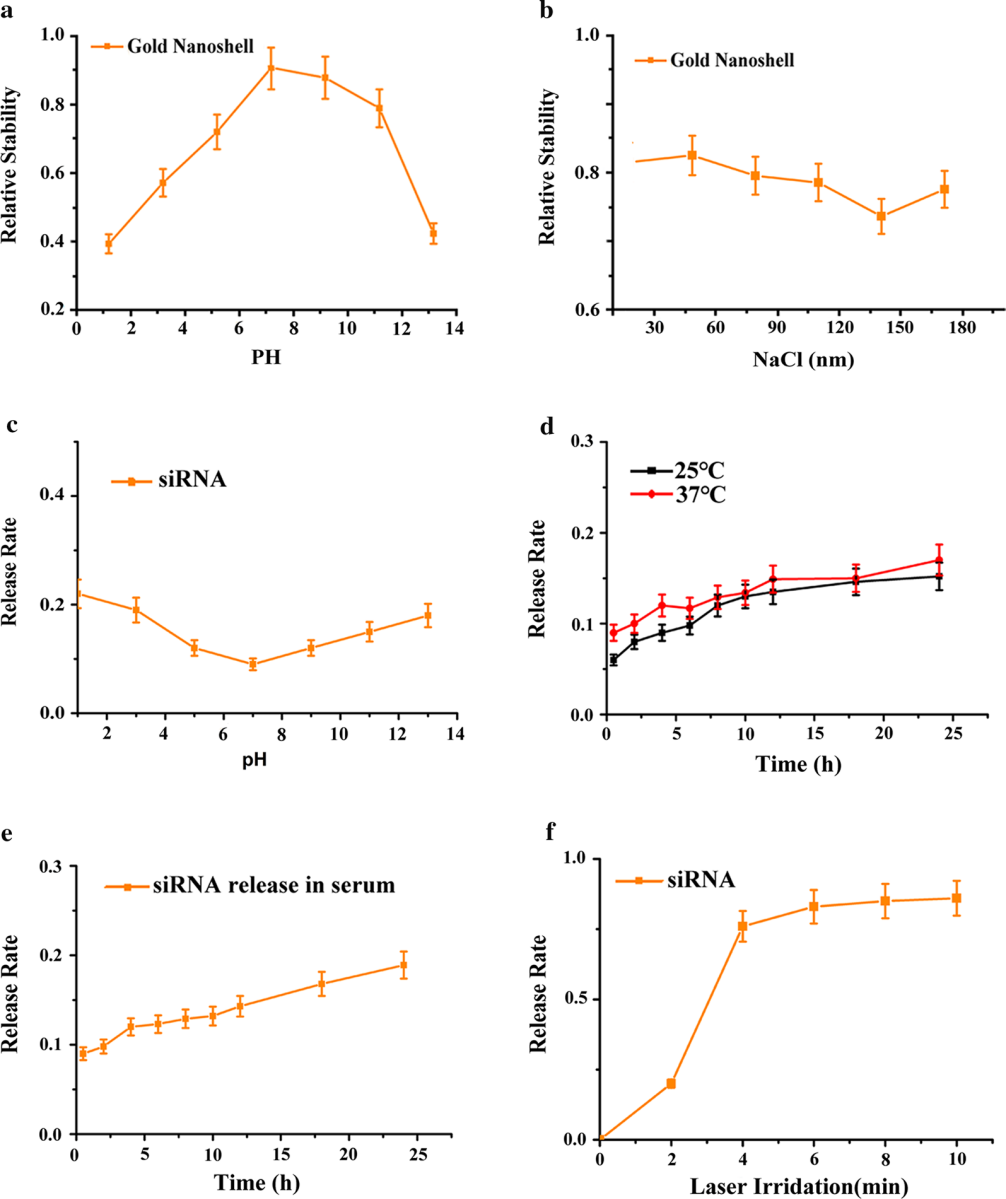
Drug releasing and stability of siRNA@CPG@Gold Nanoshell system. **a**, **b** Gold Nanoshell stability in different pH and ion strength solution. **c**–**f** siRNA release from siRNA@Gold Nanoshell in different pH situation, different NaCl concentration, in different temperature, in serum with different time and irradiated by laser with different time. Data are given as mean ± SD (n = 5). *P < 0.05

The chemical and drug release properties of Nanoshell were characterized at different pH values and different concentrations of salt solution in Fig. 2a, b. Scanning the UV absorbance value of Gold Nanoshell in the solution was scanned and its relative stability value was calculated. Our results show that the relative stability of Gold Nanoshell can reach to 70% when PH is in the range of 5–11 (Fig. 2a). Figure 2b shows the relative stability value of Gold Nanoshell in different concentrations of salt solution. Subsequent studies were carried out to investigate the stability of whole drug delivery system by analyzing the release of siRNA at different salt concentrations, pHs, temperatures and the protein presence (Fig. 2c–e). The laser-triggered release of siRNA@CPG@Gold Nanoshell as a whole were analyzed at different illumination times (Fig. 2f). We find that, within 5 min laser light exposure, there is a linear relationship between laser duration (minutes) and drug release rate (%). In addition, the light exposure of 5 min, is sufficient to trigger the release of siRNA for > 80%. These results support our objective that the release of Gold Nanoshell drug carrier could be controlled by the light illumination. It can be summarized from the results that the release rate of siRNA is low in different conditions. This unique property might benefit the proposed tumor treatment, since it is well established that the environment in vivo is full of complex. However, the release rate of siRNA still keep a low level, so it means that the drug delivery system characterized high stability except exposing under the laser irradiation.

### Properties investigation of siRNA@CPG@Gold Nanoshell

**Fig. 3:**
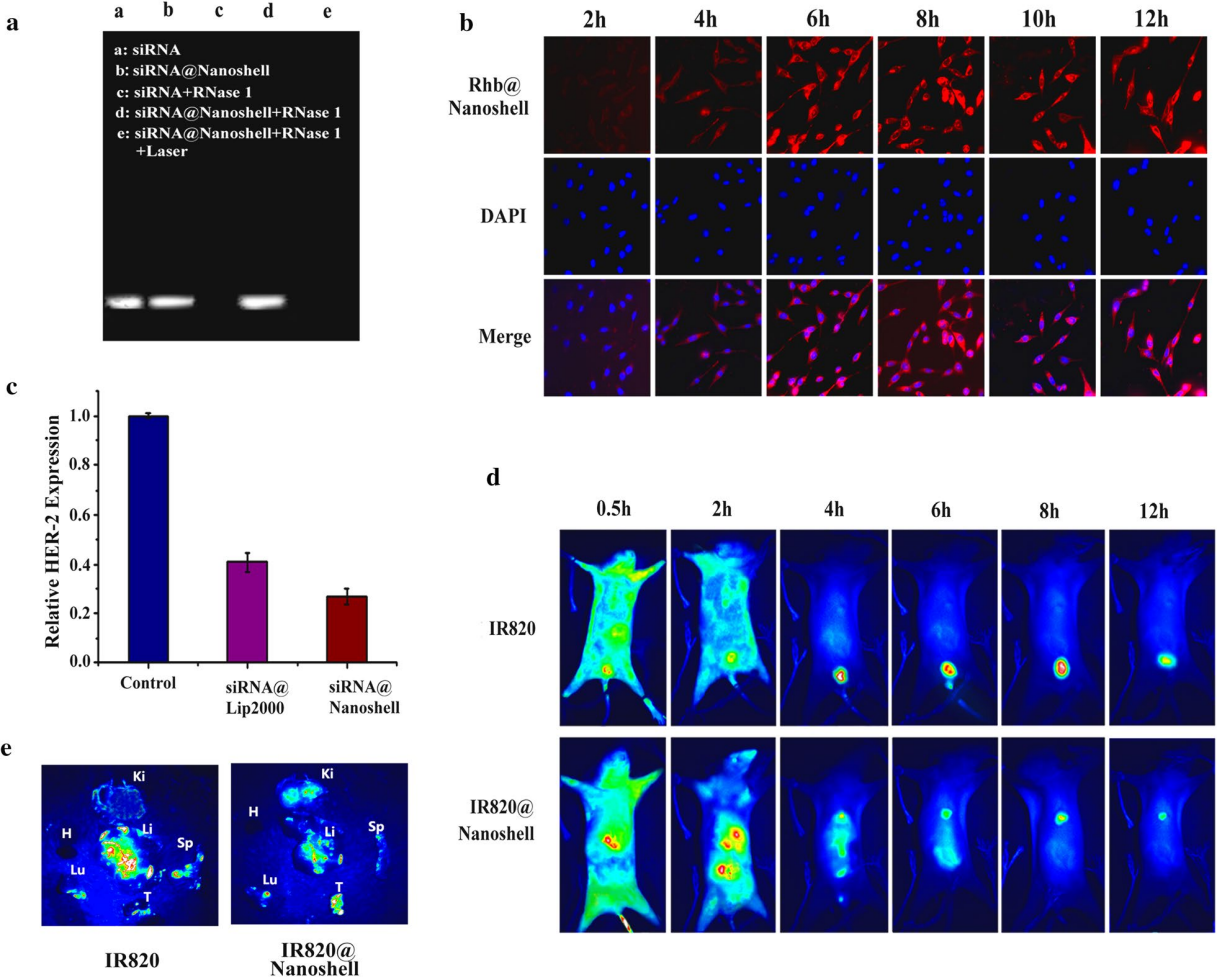
The character of siRNA@CPG@Gold Nanoshell system. **a** The nucleic acid electrophore diagram with the above conditions’ treatment. **b** The laser confocal microscopy image of siRNA@CPG@Gold Nanoshell cell uptake with different incubation time. **c** Transfection efficiency of lip2000 and Gold nanoshell with HER-2-siRNA. Data are given as mean ± SD (n = 5). *P < 0.05. **d** NIR images of IR-820 and IR-820-siRNA@CPG@Gold Nanoshell dynamics in MFC tumor-bearing mice at different times after intravenous injection. **e** The main organ distribution of IR-820 and IR-820-siRNA@CPG@Gold Nanoshell after 8 h injection

Figure 3a summarizes a nucleic acid electrophore diagram of Gold Nanoshell with the presence or absence of laser and/or RNase 1. When HER-2 siRNA is incubated with Rnase 1, no signal can be detected in the lane. While the HER-2 siRNA was attached to the Gold Nanoshell, signal could be detected after adding Rnase 1. The e lane shows that the siRNA sequence is depredated after irradiation indicate the release of siRNA from Gold Nanoshell. These results support the siRNA protective property of the Gold Nanoshell carrier. The cell uptake process of Nano-drug siRNA@CPG@Gold Nanoshell was studied (Fig. 3b). Red fluorescence is the Rhb fluorescent group which modified on the siRNA. We can see the fluorescence signal appeared in the cells within 2 h incubation and reached peak till the 6 h. It can be summarized that the siRNA@CPG@Gold Nanoshell could be taken in cells easily for taking part in the treatment function. In order to investigate the distribution and tumor targeting ability of siRNA@CPG@Gold Nanoshell in vivo, we performed near-infrared fluorescence imaging in living tumor bearing nude mice. IR-820 is a kind of hydrophilic near-infrared fluorescence dye. For the imaging we used siRNA@CPG@Gold Nanoshell to absorb the IR-820 by the electrostatic adsorption to form the IR-820-siRNA@CPG@Gold Nanoshell in this study. Figure 3c shows the comparison of transfection efficiency of Gold Nanoshell as a nano-drug carrier with transfection reagent Lip2000. The expression of HER-2 decreases significantly after transfection of HER-2 siRNA through Gold Nanoshell (27%) by RT-PCR detecting, which is lower than that of a commercial product-Lip2000 (41%) transfected samples, indicating that Gold Nanoshell which acts as an siRNA vector having a good transfection efficiency. As shown in Fig. 3d, IR-820-siRNA@CPG@Gold Nanoshell was injected into the tail vein of the MFC tumor-bearing mice, and the free IR-820 injected was used as control group. Both groups were imaged by near infrared imaging system at 0.5 h, 2 h, 4 h, 6 h, 8 h and 12 h after injection. The images show that IR-820-siRNA@CPG@Gold Nanoshell distributes in the body within 2 h. As the nano-size of the gold shell has the enhanced permeability and retention effect, the drug carrier could gradually gather to the tumor site, and its metabolism rate is slower through liver metabolism way. After 12 h, a strong fluorescence signal can still be detected in the tumor site. As for the control group, the fluorescence signal is metabolized by the kidneys. The fluorescence signal could not be detected in the organs except the bladder after injection for 4 h. In order to detect the metabolism distribution of the main organs, we sacrificed the injecting mice after 8 h. As Fig. 3e shows that the florescence signal of tumor tissue in IR-820-siRNA@CPG@Gold Nanoshell group is much stronger than the free IR-820 group and the liver is still the main metabolism organ in both groups. The result demonstrates the favorable tumor targeting ability of IR-820-siRNA@CPG@Gold Nanoshell.

### Therapy of siRNA@CPG@Gold Nanoshell in cell level

**Fig. 4:**
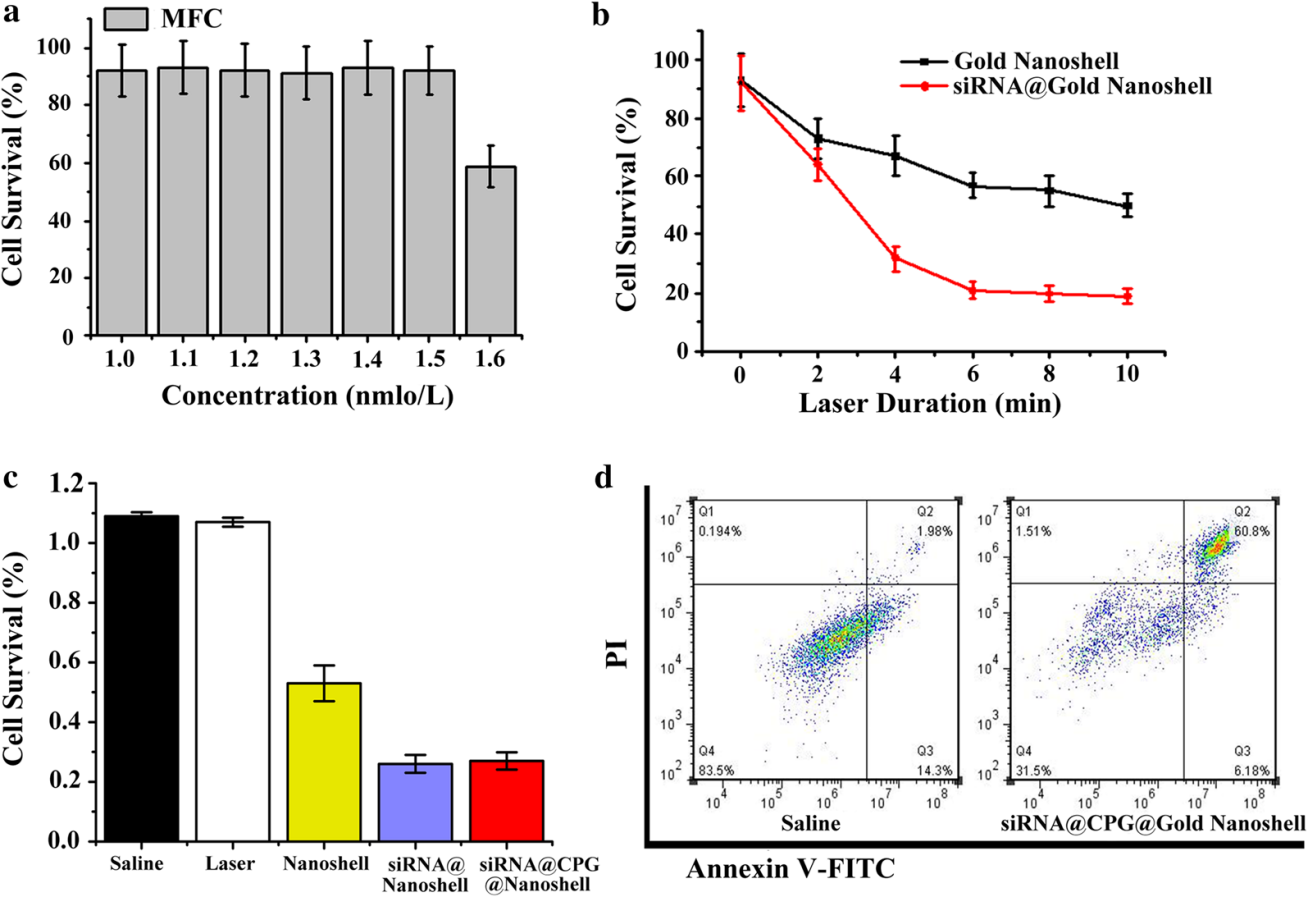
Treatment efficacy of siRNA@CPG@Gold Nanoshell in vitro. **a** Survival rate of MFC cells after incubating with different concentration of Gold Nanoshell solution. **b** Survival rate of MFC cells after incubating with Gold Nanoshell and siRNA@Gold Nanoshell with different laser irradiation time. **c** Survival rate of MFC cells treated with the above conditions. **d** Flow cytometry assay of MFC cells apoptosis rate after siRNA@Gold Nanoshell irradiation. Data are given as mean ± SD (n = 5). *P < 0.05

As it is displayed in Fig. 4a, the MFC cells incubated with Gold Nanoshell both exhibits very high survival rate which demonstrate the low cytotoxicity of this drug carrier. For investigating the optimal light irradiation time, different illumination time gradient was tested both in siRNA@CPG@Gold Nanoshell and Gold Nanoshell groups. It is showed that the cell survival rate is about 19% and 50% in the two groups after 5 min irradiation (Fig. 4b). Interestingly, we found that the laser treatment has minimal or no impact on cell survival after 5 min. It suggests that 5 min is the suitable time for the therapy. Equally important, and the treatment effect of siRNA@CPG@Gold Nanoshell group is obviously better than Gold Nanoshell group after laser irradiating. We also compared the therapeutic efficacies between single treatment and combined treatment in Fig. 4c. As shown in the diagram, the cell survival rate of siRNA@CPG@Gold Nanoshell is 26%, but siRNA@ Gold Nanoshell and Gold Nanoshell are 27%, 53% respectively. Obviously, the combined group displayed the best treatment effect. However, it should be noteworthy that the CPG modified group does not exhibit the obvious better treatment efficacy than the siRNA@Nanoshell group. Actually, it is easy to explain this phenomenon, because this test only practiced in cell level, the immunological effect causing by the CPG couldn’t be displayed. It may show the effect in the in vivo treatment.

The ability of siRNA@CPG@Gold Nanoshell to induce cell apoptosis was evaluated by a AV-PI kit (Fig. 4d). The flow cytometry results have shown that the apoptosis rate of siRNA@CPG@Gold Nanoshell reaches 66.9%. It can be seen that most cells are already in the late stage of apoptosis after treatment by siRNA@CPG@Gold Nanoshell, indicating a well treatment of siRNA@CPG@Gold Nanoshell in cell level and the combined treatment is better the single way which keep consistent with the results in Fig. 4c.

### In vivo antitumor therapeutic efficacy of siRNA@CPG@Gold Nanoshell

**Fig. 5:**
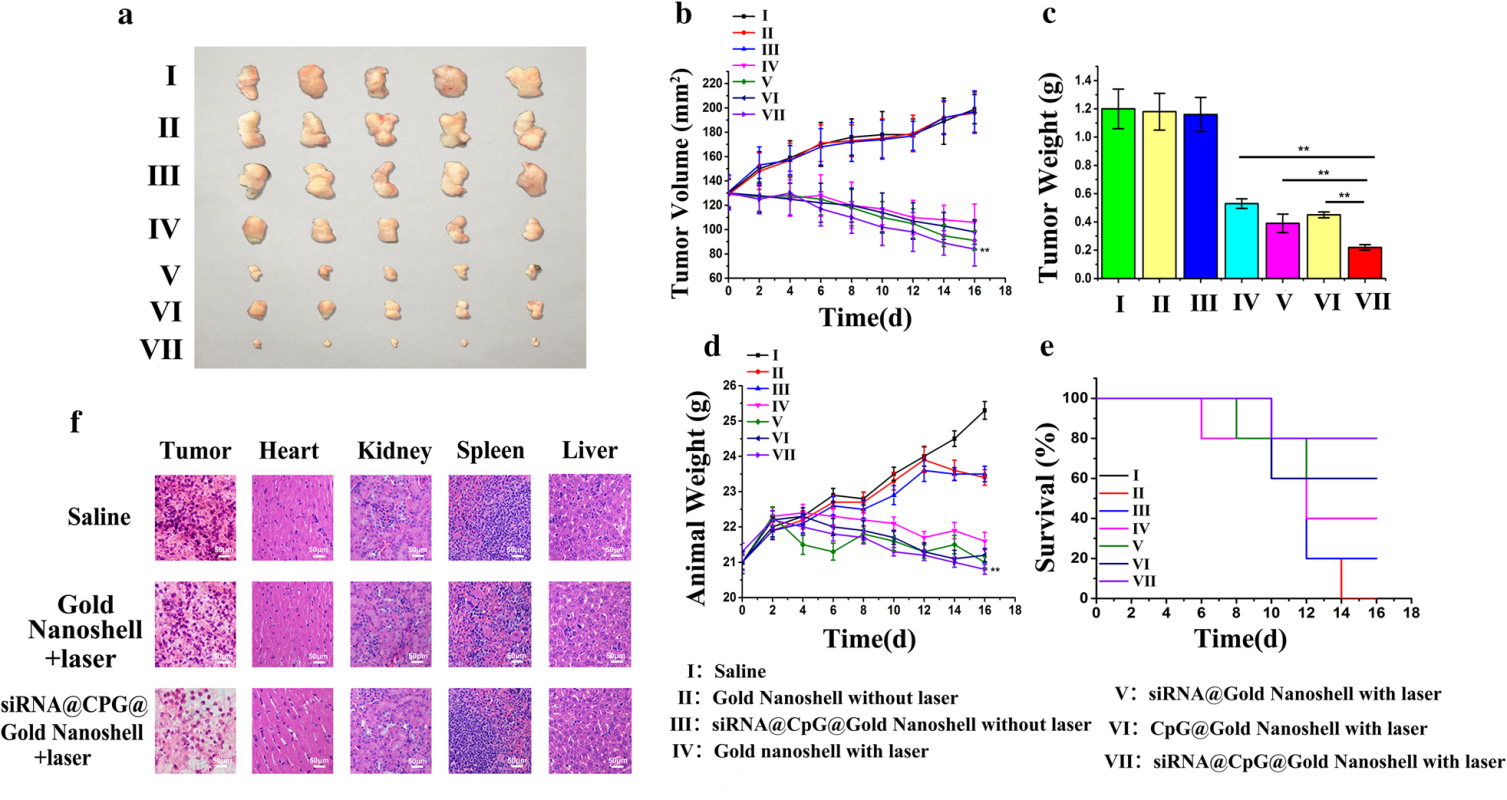
Antitumor therapeutic efficacy of siRNA@CPG@Gold Nanoshell in vivo. **a** The images of mice tumors treated with different ways as above description. **b** Tumor volume of mice-bearing MFC tumors under different treatments as above described. **c** Tumor weights of mice-bearing MFC tumors in different groups on the 16th day after injection. **d** Body weights of mice bearing MFC tumors in different groups. **e** The 16-day survival rates of mice after different administration. **f** H&E stained images of tumor, heart, liver, spleen and kidney sections collected from different treatment groups. Data are given as mean ± SD (n = 5). *P < 0.05

The in vivo antitumor efficacy of siRNA@CPG@Gold Nanoshell (testing groups: Saline, Gold Nanoshell with laser, siRNA@CPG@Gold Nanoshell without laser, Gold Nanoshell with laser, siRNA@ Gold Nanoshell with laser, CpG@ Gold Nanoshell with laser, siRNA@CPG@Gold Nanoshell with laser) was further evaluated in a mice model. We measure the effect of different treatment on the tumor growth rate, survival rate and the body weight of the mice. As shown in Fig. 5a–c the tumor of saline -treated mice grow faster than that of the other treatment groups, and the siRNA@CPG@Gold Nanoshell treatment exhibits the optimal inhibition effect of tumor growth. It is noted that the growth of MFC tumor is inhibited with a rate of 82% after the administration of siRNA@CPG@Gold Nanoshell which is higher than siRNA@Nanoshell (67.5%). The index of synergetic effect was calculated: tumor weight rate of W_V/I_ *W_VI/I_ = 12%, and tumor weight rate of W_VII/I_ was about 18%. So it is suggested that the synergetic treatment worked well. Furthermore, the animal weight of mice-bearing MFC tumors in siRNA@CPG@Gold Nanoshell group almost maintain stably compared with the free Gold Nanoshell and siRNA@Nanoshell (Fig. 5d). The 22-day survival rate of mice in the RGD @ DOX @ siRNA @ Gold Nanoshell group was 80% which obviously higher than siRNA@Nanoshell (40%) groups, whereas all the mice in saline group died on the 16th day (Fig. 5e). From the results in Fig. 5, it is easy to see that the efficacy of combined treatment is obviously better than the single one and the synergistic effect is realized by the siRNA@CPG@Gold Nanoshell drug carrier system.

To further investigate the biological toxicity on the major organs of mice and anti-tumor effects of the above treatments, histological examination was conducted. Our results are shown in Fig. 5f that the number of tumor cells in the saline-injected group are significantly more than that in the other two groups, and the tumor cells are in a dense mitotic condition, which suggested that the tumor cells would continue to speed up the proliferation process. And the examination of tumor tissues in siRNA@CPG@Gold Nanoshell group revealed more pronounced pathological changes than other groups. It is to say that the tumor treatment efficacy of siRNA@CPG@Gold Nanoshell is better than siRNA@Nanoshell groups in this experiment. At the same time, it is exhibited that no obvious pathological changes in other major organs which suggests the safety of siRNA@CPG@Gold Nanoshell drug carrier system.

### Immune responses after siRNA@CPG@Gold Nanoshell based PTT

**Fig. 6:**
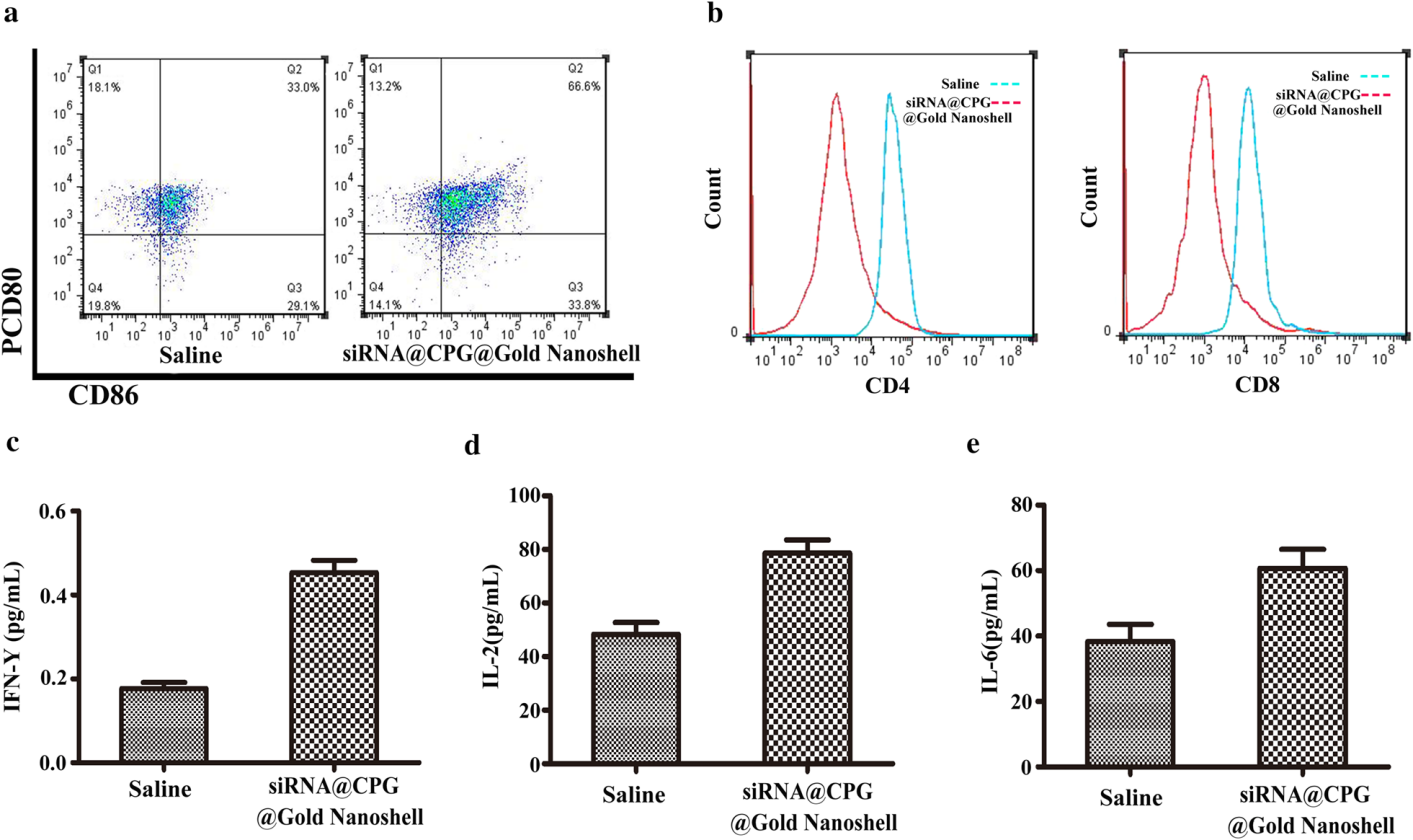
Immune responses after siRNA@CPG@Gold Nanoshell-based PTT. **a** DC maturation induced by siRNA@CPG@Gold Nanoshell-based PTT on mice-bearing MFC tumors. Cells in the tumor-draining lymph nodes were collected 72 h after various treatments for assessment by flow cytometry after staining with CD80 and CD86. **b** Flow cytometry assay of CD4 + and CD8 + T cells in the tumor-draining lymph nodes after siRNA@CPG@Gold Nanoshell-based PTT. C,D,E, Cytokine levels (IFN-γ, IL-2, IL-6) in sera from mice isolated at 72 h post different treatments siRNA@CPG@Gold Nanoshell-based PTT. Data are given as mean ± SD (n = 5). *P < 0.05

The function of CpG in activating the mature myeloid dendritic cells was assayed by the flow cytometric. The analysis of flow cytometric showed that the amount of mature myeloid dendritic cells (CD80^+^CD86^+^) in tumors of mice injected with siRNA@CPG@Gold Nanoshell (66.6%) increased obviously more than saline group (33%) (Fig. 6a). In addition, siRNA@CPG@Gold Nanoshell with laser treatment induce the greatest number of mature myeloid dendritic cells migrating to tumor-draining lymph nodes among all the groups. Collectively, these results indicate that photothermal ablation and the photothermally triggered formation of Gold nanoshell system have an obviously effect on the activation of myeloid dendritic cells following siRNA@CPG@Gold Nanoshell injection with laser treatment.

Cytotoxic T lymphocytes which have the ability to eradicate cancer cells in vivo could be a measurement on the immunotherapy effect. The amount of effector CD8+ and CD4 + T cells is considered as an indicator for immune responses in vivo. So, here, the assessment of CD8+ and CD4 + T cells was conducted. The results exhibit that siRNA@CPG@Gold Nanoshell with laser treatment could both increase the amount of CD8+ and CD4 + T cells in tumor-draining lymph nodes (Fig. 6b). It is worth noting that siRNA@CPG@HAuNs with laser treatment elicited systemic immunity, as evidenced by the significantly increased amount of IFN-γ which secreted by the CD8 + T cells in tumor, comparing with the saline control. In addition, the treatment also leads the levels of IL-2 and IL-6 obviously increased in tumor tissue (Fig. 6c–e). All of the above results suggest that the combined treatment with photothermal therapy and immunotherapy could additional activate the systemic immunity to eradicate tumor in vivo.

## Conclusion

In this study, we established a drug carrier system siRNA@CPG@Gold Nanoshell which focus on the treatment of the gastric cancer MFC. From the results, the drug carrier system shows many good abilities such as the high drug uploading rate, control release and tumor targeting et al. The combination of gene therapy, thermal therapy and immunotherapy exhibit the obviously well treatment effect on MFC tumor cells both in vitro and in vivo. In a conclusion, this study provides a promising method for the gastric cancer treatment.

## Data Availability

The data and material in this paper are available.
